# F150. OVERESTIMATING ENVIRONMENTAL VOLATILITY INCREASES SWITCHING BEHAVIOR AND IS LINKED TO ACTIVATION OF DORSOLATERAL PREFRONTAL CORTEX IN SCHIZOPHRENIA

**DOI:** 10.1093/schbul/sby017.681

**Published:** 2018-04-01

**Authors:** Lorenz Deserno, Rebecca Boehme, Christoph Mathys, Teresa Katthagen, Jakob Kaminski, Klaas Enno Stephan, Andreas Heinz, Florian Schlagenhauf

**Affiliations:** 1 Max Planck Institute for Human Cognitive and Brain Sciences; 2 Center for Social and Affective Neuroscience, Linköping University; 3 Scuola Internazionale Superiore di Studi Avanzati (SISSA); 4 Charité – Universitätsmedizin Berlin; 5 University of Zurich & ETH Zurich

## Abstract

**Background:**

Reward-based decision-making is impaired in schizophrenia, as reflected by increased switching between choices. The underlying cognitive mechanisms and associated neural signatures remain unknown. Reinforcement learning (RL) and hierarchical Bayesian learning account for this behavior in different ways. We hypothesized that enhanced switching during flexible reward-based decision-making in schizophrenia relates to higher-order beliefs about environmental volatility and examined the associated neural signatures.

**Methods:**

46 medicated schizophrenia patients and 43 controls underwent a reward-based decision-making task requiring flexible behavior to changing action-outcome contingencies during functional Magnetic Resonance Imaging (fMRI). Computational modeling of behavior was performed, including RL and the Hierarchical Gaussian Filter (HGF). The estimated learning trajectories informed the analysis of fMRI data.

**Results:**

A three-level HGF accounted best for the observed choice data and revealed a heightened prior belief about environmental volatility and a stronger influence of volatility on lower-level learning of action-outcome contingencies in schizophrenia. This finding was replicated in an independent sample of unmedicated patients. Beliefs about environmental volatility were reflected by higher activity in dorsolateral prefrontal cortex (dlPFC) of patients compared to controls.

**Discussion:**

This study suggests a mechanistic explanation for instable behavior in schizophrenia: patients inferred the environment as being too volatile and thus overestimated environmental changes, leading to maladaptive choice switching. Our data suggest enhanced dlPFC activity related to beliefs about environmental volatility as a neural learning signature of instable behavior. Such detailed ‘computational phenotyping’ may provide useful information to dissect clinical heterogeneity and could improve prediction of outcome.

